# Phonetic-phonological performance of typical younger and aged adults from Brazil’s capital city

**DOI:** 10.1590/1980-57642020dn14-030012

**Published:** 2020

**Authors:** Tuany Aquino Nogueira, Liara Dias da Silva, Maysa Luchesi Cera

**Affiliations:** 1 Faculdade de Ceilândia, Universidade de Brasília, Brasília, DF, Brazil.

**Keywords:** aging, language, speech, phonetics, aged, health of the elderly, envelhecimento, idioma, fala, fonética, idoso, saúde do idoso

## Abstract

Given the sociodemographic diversity in Brazil, it is fundamental to understand the speech performance of a sample from the Brazilian capital. The repetition task can assess phonological and motor-phonetic planning. Previous studies found phonological-phonetic performance of speakers to be associated with education, age, and other demographic factors. Objectives: To compare the phonetic-phonological performance for speech of younger and aged adults in the capital of Brazil, Federal District (FD); to compare FD performance against national normative means based on São Paulo; to determine the association of phonetic-phonological agility with sociodemographic, cognitive, and neuropsychiatric variables for the sample. Methods: Cross-sectional study. A total of 60 volunteers from the FD, comprising 30 older adults and 30 younger ones, were stratified by education into two subgroups: 2‒7 years and ≥8 years of education. Data on age, educational level, and socioeconomic status were collected. The Verbal Agility subtest of the Boston Diagnostic Aphasia Examination was applied to assess phonetic-phonological performance. Results: No statistically significant difference in performance for verbal agility was found between aged and younger adults from the FD. There was a statistically significant difference in the phonetic-phonological performance of the FD sample compared with the Brazilian normative mean values. Cognitive and socioeconomic variables were associated with verbal agility. Conclusions: In the capital of Brazil, economic status, age, education, and cognitive variables were associated with verbal agility performance, despite there being no difference in phonetic-phonological performance between younger and aged adult groups. Regional differences in phonetic-phonological performance were also evident.

## RESUMO.

Diante da diversidade sociodemográfica do Brasil, é fundamental compreender o desempenho de fala de uma amostra da capital do país. O teste de repetição pode avaliar o planejamento fonológico e fonético-motor. Estudos prévios mostram que o desempenho fonético-fonológico de falantes foi associado à escolaridade, à idade e a outras variáveis demográficas. Objetivos: Comparar o desempenho fonético-fonológico da fala de idosos e adultos jovens da capital do Brasil, Distrito Federal (DF); comparar o desempenho da amostra do DF com as médias normativas nacionais, obtidas em São Paulo; e verificar a associação da agilidade fonético-fonológica da nossa amostra com as variáveis sociodemográficas, cognitivas e neuropsiquiátricas. Métodos: Estudo transversal. Foram selecionados 60 voluntários do DF, 30 idosos e 30 adultos jovens, subdivididos em dois grupos conforme a escolaridade, de 2 a 7 anos e de 8 anos ou mais de estudo. As características etárias, de escolaridade e de classe econômica foram coletadas. O subteste de agilidade verbal do Teste de Boston para Diagnóstico das Afasias foi aplicado para avaliação do desempenho fonético-fonológico. Resultados: O desempenho de agilidade verbal dos idosos não apresentou diferença estatisticamente significante em relação aos adultos jovens do DF. Houve diferença estatisticamente significante ao se comparar o desempenho fonético-fonológico da amostra do DF com as médias normativas brasileiras. As variáveis cognitivas e socioeconômicas estiveram associadas à agilidade verbal. Conclusões: Na capital do Brasil, as variáveis econômicas, etárias, de escolaridade e cognitivas estiveram associadas à agilidade verbal, apesar da ausência de diferença do desempenho fonético-fonológico entre adultos jovens e idosos. Houve diferenças de desempenho fonético-fonológico conforme a região do país.

## INTRODUCTION

According to population projections by the Brazilian Institute of Geography and Statistics (IBGE), the proportion of elderly in the country’s capital city, the Federal District (FD), is set to rise from 11% in 2019 to 33% by 2060.^1^ In view of the growing elderly population and consequent prioritizing of promotion and prevention of elderly health, enshrined in Law 8.842 of 1994,^2^ understanding the changes associated with the aging process is paramount.

Phonological working memory abilities decline with age.^3^ However, high-educated Brazilian elderly have shown better cognitive performance^4^ and motor speed has predicted deficits in the cognition of individuals with neuropsychiatric disorders,^5^ suggesting a relationship between cognitive-communicative performance and sociodemographic and neuropsychiatric characteristics.

Phonological planning is a linguistic-symbolic function responsible for the sequential selection and combination of phonemes, while phonetic-motor planning is responsible for transforming phonemes into action, a strategy for the motor target.^6^ The repetition task can assess both phonological and motor-phonetic speech planning. These communication processing stages can be assessed by the verbal agility subtest from the Boston Diagnostic Aphasia Examination (BDAE).^7^ In this subtest, the performance of speakers of Brazilian Portuguese and English was found to be associated with education.^8^ ^,^ ^9^ However, in contrast to a North American study,^8^ no statistically significant difference in verbal agility was found between different age groups in a Brazilian study.^9^

It is noteworthy that the normative data for the verbal agility subtest used throughout Brazil were collected in São Paulo (SP), southeastern region of the country.^9^ As previously reported, sociodemographic factors influence cognitive performance.^4^ ^,^ ^9^ Given the sociodemographic diversity in Brazil, it is essential to understand the phonetic-phonological speech performance of a sample from the Brazilian capital, and its relationship with the participants’ sociodemographic characteristics.

Therefore, the objective of this study was to compare the verbal agility performance of younger and aged adults from the FD, capital of Brazil. The study hypothesis is that the verbal agility performance of younger adults is superior to that of aged ones. The study also compared the verbal agility performance of the FD sample with that of normative means for the Brazilian population and observed that it is different from those obtained in other regions of the country. The study also aimed to determine the association between phonetic-phonological performance and sociodemographic, cognitive, and neuropsychiatric variables. The hypothesis is that these variables are associated with verbal agility.

## METHODS

The study was approved by the Research Ethics Committee of the University, under CAAE 56190716.0.0000.0030 and permit No. 1.657.122. All participants signed the Informed Consent Form, drafted in accordance with Resolution No. 466/2012 of the Brazilian National Health Council, Ministry of Health.

The study sample comprised 60 volunteers from the Brazilian capital, stratified by age into 2 groups: aged adults (≥60 years of age) and younger adults (19‒39 years of age). Fifteen volunteers from each group had 2‒7 years of education, whereas 15 had ≥8 years of education.

This study is part of a larger project by the FortaleçaMente Group, involved in education, research, and extension activities on communication involving adults and elderly from the FD. The inclusion and exclusion criteria applied to participants have been published elsewhere by the FortaleçaMente Group.^10^ Inclusion criteria were: having a typical stable state of health, with no history of psychiatric or neurologic diseases, being right-handed, and having lived in the FD for 10 years or longer. Exclusion criteria were: having a prior or current history of alcoholism or illegal drug use; having visual or hearing deficits or being in use of a hearing aid or poorly adapted dental prosthesis that could negatively affect performance on the tasks performed; and having below expected results on screening tests for cognition and depression.

The phonetic-phonological assessments were carried out in a quiet space at the volunteers’ homes, institutions or other venue of their choice. As described by Cera and Silva,^10^ information on age, gender, education, and economic status were collected, according to the Brazilian Economic Classification Criteria,^11^ to determine socioeconomic levels; and assessments were performed using the Mini-Mental State Exam (MMSE)^4^ ^,^ ^12^ for global cognitive screening; and by the Geriatric Depression Scale (GDS)^13^ to identify signs of depression. These instruments are described below:

- Brazilian Association of Research Companies (*Associação Brasileira de Empresas de Pesquisa -* ABEP):^11^ questionnaire collecting data on items in household, householder education, and access to public utility services, to provide an estimate of household income, scored on a scale of 0 to 100, with greater scores indicating higher economic class;
- MMSE:^4^ ^,^ ^12^ participants’ scores exceeded the cut-off values for the Brazilian population published by Bertolucci et al.,^4^ of 18 points for education of 1‒7 years, and 26 for ≥8 years;
- GDS:^13^ the short form of the scale with a maximum score of 15 was used, in which scores ≥5 points suggest depression. All study participants scored >5 points.

In addition, the Verbal agility subtest of the BDAE^7^ was applied for the phonetic-phonological assessment. In this test, subjects are instructed to repeat words as quickly as possible and the number of repetitions in 5 seconds is registered. The test stimuli for the Brazilian version are as follows: 1) *vovó-vovó*, 2) *tic-tac*, 3) *pula-pula*, 4) *saúde-saúde*, 5) Filomena, 6) *ponta direita*, and 7) *lagartixa*. The sequence of repetition for each stimuli from the subtest is scored with 0, 1 or 2 points according to pre-established repetition bands, with 2 points assigned for producing a greater number of correct repetitions of the stimuli; 1 point for lower numbers of correct repetitions; and 0 points for correct repetitions below the minimum number expected. Total score ranges from 0 (worst performance) to 14 (best performance) points. The Brazilian normative mean values collected in SP are 12.6 points for the overall sample, 11.9 for subjects with educational level of 1‒8 years, and 13.0 for those with >9 years of education.^9^

### Statistical method

The Kolmogorov-Smirnov determined the data distribution, defining the selection of statistical tests for the inferential analysis. The IBM SPSS Statistics 22 software was used for statistical tests.

Fisher’s exact and Mann-Whitney tests were used to compare sociodemographic, neuropsychiatric, and cognitive variables between the groups.

The Mann-Whitney test was used to compare verbal agility performance of the younger and aged adults.

Student’s t-test was employed to the compare phonological performance of the FD sample against Brazilian normative mean values*.*

Spearman’s correlation coefficient was used to analyze the association of verbal agility with sociodemographic, neuropsychiatric, and cognitive variables.

A p value of 0.05 was adopted to indicate statistical significance.

## RESULTS

A total of 65 volunteers from the FD took part in the study. Five subjects were subsequently excluded for scoring lower than Brazilian means on the cognitive screening and depression tests. The proportion of females in each group ranged from 87 to 90%. Mean score on the ABEP was 31.2 (9.8) for younger adults and 26.8 (7.8) for older adults, both classified with social economic stratum B2. Mean age of the older adults was 66.8 (4.6) years and younger adults was 28.7 (7.9) years; mean education was 7.6 (3.7) years and 8.2 (4.1) years; mean time residing in the FD was 47.6 (9.5) years and 24.0 (7.5) years; GDS score was 1.5 (2.0) and 2.9 (2.3); and MMSE score was 26.2 (0.2) and 26.6 (0.8), respectively. There was no statistically significant difference in economic class, education or cognition between the two age groups, except for depression screening score, which proved lower among the younger group.^10^

Regarding findings of the present study for phonetic-phonological aspects, Table 1 presents a comparison of the performance by the groups of younger and aged adults from the FD, revealing no statistically significant difference for age.

**Table 1.** Comparison of phonetic-phonological performance of younger and aged adults from the Brazilian capital, Federal District, using the Mann-Whitney test.

Groups	U	p-value
Aged *versus* younger adults	397.50	0.420
Low-educated aged *versus* younger adults	88.00	0.325
High-educated aged *versus* younger adults	67.50	0.061

A comparison of verbal agility performance of the FD volunteers against Brazilian normative mean values for SP is shown in Table 2. There was a statistically significant difference between the groups, with the FD sample exhibiting worse verbal agility performance.

**Table 2.** Comparison of verbal agility performance of Federal District volunteers with Brazilian normative mean values for São Paulo.

Groups	Mean and standard deviation for FD*	Mean and standard deviation for SP+	Difference between means	95%CI	Student’s t-test	p-value
Volunteers (n=60)	11.2 (1.7)	12.6 (1.7)	-1.02	-1.46 to -0.57	-4.55	0.001
Low-educated group (n=30)	11.2 (1.8)	11.9 (2.0)	-0.73	-1.39 to -0.08	-2.28	0.030
High-educated group (n=30)	12.00 (1.6)	13.0 (1.3)	-1.00	-1.60 to -0.40	-3.38	0.002

95%CI: 95% confidence interval; FD*: Federal District; SP+: São Paulo.

Results of the correlation between verbal agility performance and sociodemographic, neuropsychiatric, and cognitive variables are given in Table 3. The association of age, education, economic status, and cognitive performance with verbal agility proved to be statistically significant. For the overall sample, verbal agility performance correlated with education, socioeconomic class, and score on the screening test. In the low-educated group, no association was found between speech production and the variables studied. For the high-educated group, verbal agility performance was correlated with both age and education.

**Table 3.** Correlation between verbal agility performance of Federal District volunteers and sociodemographic variables.

GROUPS	Statistics	Age	Education	Economic status	Depression	MMSE
Federal District volunteers (Aged and Younger adults) (n=60)	Coefficient of correlation	-0.21	0.41	0.28	0.23	0.27
	p-value	0.105	0.001	0.028	0.080	0.038
Low-educated group (n=30)	Coefficient of correlation	0.03	0.27	0.21	0.30	0.05
	p-value	0.877	0.156	0.259	0.110	0.786
High-educated group (n=30)	Coefficient of correlation	-0.52	0.40	0.24	0.16	0.24
	p-value	0.003	0.030	0.209	0.396	0.199

MMSE: Mini-Mental State Exam.

## DISCUSSION

The main finding of this study was that there was no statistically significant difference in phonetic-phonological performance between younger and aged adults from the FD - capital of Brazil (Table 1) -, but the performance of the FD sample was worse than the normative national mean, derived from SP data (Table 2). Also, verbal agility was associated with the sociodemographic variables and with scores on the cognitive screening tests (Table 3). These findings are discussed below.

The results for the comparison of verbal agility performance of younger and aged adults corroborate the Brazilian study, which observed similar phonetic-phonological performance across the three age groups studied,^9^ yet differed from a study conducted in another country.^8^ Decline in phonological processing with age has been observed in other studies.^14^ ^,^ ^15^ ^,^ ^16^ The difference compared to a previous study assessing verbal agility^8^ was likely due to sociodemographic disparities between the countries or differences in the languages and their variations. In the present study, verbal agility was assessed using a repetition task, *i.e*., phonological output processing, whereas other studies which detected impairments in phonological processing with aging assessed input processing^14^ ^,^ ^16^ or utterances using the naming task.^15^ Thus, the differences in the present results relative to those of other studies might be related to the stage of language processing assessed, input or output; to the method of assessing phonological processing, repetition or naming; or to sociodemographic and linguistic differences.

During the aging process, compensatory activities are active in conjunction with neural plasticity.^17^ It is therefore believed that, for verbal agility, Brazilians use compensatory strategies to maintain phonetic-phonological performance, despite the effects of aging. This notion is supported by the present study and by a previous investigation carried out in another part of the country,^9^ both of which reported similar output phonetic-phonological processing results. Moreover, in the present study, the similarities in sex, education, economic class, and cognitive performance for the groups studied may also have contributed to the absence of difference in verbal agility between younger and aged adults,^10^ given that age was the only variable that differed between the groups.

Sociodemographic disparities can be found in different regions of Brazil - a country with major socioeconomic and cultural diversity. Therefore, phonetic-phonological performance of volunteers from FD were compared to national normative averages for SP.^9^ The volunteers from the Brazilian capital had worse performance (Table 2), corroborating the findings regarding the association of sociodemographic variables with cognitive,^18^ ^,^ ^19^ ^,^ ^20^ language,^14^ ^,^ ^15^ ^,^ ^16^ ^,^ ^21^ ^,^ ^22^ and specifically with verbal agility performance.^8^ ^,^ ^9^ Differences in verbal agility between the samples from different regions of Brazil may also be related to linguistic variations, especially in light of the previous observation that speakers from SP city have regional variations in speech that differ from other regions of the country.^23^ In addition, it is important to bear in mind that most elderly in the FD were originally from other regions of the country, thus conferring more diverse speech characteristics. Moreover, results showed that average time living in the FD was 48 years for aged adults and 24 years for younger ones, factors which may further the systematization of speech characteristics of the population from the country’s capital. This language variation typical of the FD was previously reported,^24^ although the study in question did not assess speech rate in this region of the country. Based on the difference between the Brazilian normative means and those found in the present study, it can be concluded that speakers from the FD have lower verbal agility performance, suggesting a slower speech rate compared to speakers from SP. For neurologic disorders to be properly diagnosed, regional phonetic-phonological characteristics should be taken into account. The present study reports the verbal agility performance of Brazilians from the FD, capital of Brazil since 1960. Previous Brazilian studies on verbal agility have been conducted in the SP region in both typical speakers^9^ and individuals with neurological disorders.^25^ Interestingly, the mean age of the overall sample from the FD was 47.7 (20.2) years, almost identical to that of the SP sample, of 47.2 (17.6) years.^9^ It therefore follows that normative differences for the FD and SP found in the present study were not attributed to participants’ age.

In addition, the association between phonetic-phonological performance and sociodemographic, cognitive, and neuropsychiatric variables is explored below.

Age was associated with verbal agility in the high-educated group (Table 3). Thus, this study suggests that the areas responsible for linguistic-phonological and motor-phonetic processing remain activated in a similar fashion in both younger and aged adults. This holds true, except for higher educational level, in which case better verbal agility performance is associated with lower age. With regard to the neuroanatomical substrates of acquired phonological and phonetic disorders, Ripamonti et al. listed the following regions as responsible for phonetic processing: left central operculum, left inferior frontal gyrus, left precentral gyrus, left insula, left parietal operculum, left supramarginal gyrus, left supplementary motor cortex, left frontal operculum, and left putamen.^26^ The regions described as responsible for phonological processing were: posterior portion of the left superior temporal gyrus, left supramarginal gyrus, Heschl’s gyrus, left temporal plane, posterior portion of the left middle temporal gyrus, and left parietal operculum. ^26^ Thus, differences in the functioning of the regions responsible for linguistic-phonological and motor-phonetic processing with greater length of formal study may have influenced the results found in this study of better verbal agility performance associated with lower age in high-educated subjects.

The result showing an association of education with verbal agility, where the greater the verbal agility, the longer the period of formal study (Table 3), corroborates studies on verbal agility measured using the BDAE^8^ ^,^ ^9^ and faster cognitive processing speed for higher educated individuals.^27^

The present study revealed that, the greater the educational level, the stronger the association of phonetic-phonological performance with age and education. This result corroborates the study by Rentz et al., which suggested a neuroprotective effect and less vulnerability to the neurotoxic effects among subjects with greater cognitive reserve.^28^ Cognitive reserve is determined by various factors, including socioeconomic and educational,^19^ while sociodemographic aspects influence cognitive performance.^18^ ^,^ ^20^ In a study involving participants from Latin American countries, age influenced language performance in only some countries, leading to norms adjusted for age in just some of these regions, while the effect of education on language performance was seen in all countries studied.^22^ In Brazil, the influence of age and education on language performance was found in many different regions.^21^

It is interesting to note that, besides education, economic status and cognitive score were also associated with verbal agility performance in the sample of 60 volunteers assessed (Table 3). Scarmeas et al. argued that occupational and educational aspects are life experiences that may allow some people to better cope with cognitive changes and that challenging experiences may attenuate neurodegenerative processes.^19^ Although conducted in a society with relatively low income inequality, the results of the study by Nishizawa et al. suggest that socioeconomic status in childhood may be an element of cognitive reserve, with important implications for late-life subjective memory complaints.^29^ Thus, reports in the literature are consistent with our conclusions, confirming that sociodemographic factors are associated with cognitive abilities, specifically phonetic-phonological performance.

The present study has some limitations that deserve to be highlighted, such as the fact that most volunteers were female. Consequently, generalizing these results for men should be done with caution. However, previous studies observed no differences between men and women for speech fluency.^30^ Furthermore, the present study analyzed the quantitative performance for phonetic-phonological processing, based on scores on the verbal agility subtest. However, in clinical practice, in addition to quantitative analyses of performance in the verbal agility subtest, it is fundamental that the qualitative analyses of phonetic symptoms be differentiated from phonological ones, as per the differential diagnosis of aphasia and apraxia of speech.^31^ ^,^ ^32^

Therefore, study results confirmed no difference in agility between the younger and aged adults from the FD. The FD sample, however, had statistically worse phonetic-phonological performance relative to Brazilian normative means. Lastly, the sociodemographic and cognitive factors were associated with verbal ability performance.
